# Exposure to particulate matter (PM_2.5_) and volatile organic compounds (VOCs), and self-reported health symptoms among fish smokers: A case study in the Western Region of Ghana

**DOI:** 10.1371/journal.pone.0283438

**Published:** 2023-03-24

**Authors:** Gifty Mensah Obeng, Simon Appah Aram, Daniel Agyei, Benjamin M. Saalidong

**Affiliations:** 1 Department of Environmental Science, University of Cape Coast, Cape Coast, Ghana; 2 College of Safety and Emergency Management Engineering, Taiyuan University of Technology, Taiyuan, People’s Republic of China; 3 Department of Environmental and Natural Resource Management, Presbyterian University, Ghana, Abetefi, Ghana; 4 Department of Geoscience and Engineering, Taiyuan University of Technology, Taiyuan, People’s Republic of China; Satyawati College, University of Delhi, INDIA

## Abstract

The study aimed to assess the concentrations of particulate matter (PM_2.5_) and volatile organic compounds (VOCs) produced from the burning of biomass fuel from the smoking of fish. It also sought to determine the proportion of fish smokers reporting health symptoms associated with exposure to these pollutants. A cross-sectional study was conducted among fish smokers at Abuesi in the Western Region of Ghana. Systematic sampling was employed to choose 60 smokehouses for PM_2.5_ and VOC monitoring. A total of 434 fish smokers were also randomly sampled for the study. Measurements were taken at indoor, outdoor and control locations. The highest concentration of PM_2.5_ was recorded in the indoor environment. The mean concentration of PM_2.5_ between the indoor and control environment was significantly different unlike between the outdoor and control environments. The concentration of VOCs systematically varied across indoor, outdoor and control locations. The most reported disease symptoms were eye infection, cough, and headaches. There was a strong positive association between the number of years spent smoking fish and the frequency of eye problems reported by fish smokers. The study demonstrated that fish smokers inside the smokehouse or smoking rooms are exposed to higher PM_2.5_ and VOC levels which are detrimental to their health. There is therefore the need for further studies to explore other energy sources which may have a lesser negative effect on human health.

## Introduction

Clean air is considered to be a basic necessity for human health and well-being. Globally, many people obtain their daily domestic energy from solid fuels such as wood, coal and crop residues [1]. The extensive use of solid fuels for domestic cooking is estimated to be the main source of indoor air pollution worldwide [2, 3]. When biomass fuel is incompletely burnt, harmful particles such as particulate matter, carbon monoxide and nitrogen dioxide are released. These harmful particles can cause severe harm to human health [4, 5]. These pollutants are tiny in size and can easily cross the alveolar-capillary barrier and enter deeper parts of the lungs [6]. Some epidemiological studies have shown a strong association between particulate matter exposure with adverse health effects such as lung cancer, heart failure, asthma, and mortality [7, 8].

The concentration of pollutants from biofuels such as carbon monoxide and particulate matter varies with kitchen type and fuel type. According to Sidhu et al. [9], the highest concentrations of pollutants from biomass fuel have been observed in kitchens using cow-dung cakes and agriculture residues as fuel. Embiale et al. [10] have also shown that the use of clean stoves or improved stoves during cooking minimizes air pollutants generation as compared to traditional stoves. In most developing countries, cooking with biomass fuels is generally done on traditional cookstoves which are usually unvented stoves consisting of simple 3 rocks arrangements, a U-shaped hole in a block of clay, or a pit in the ground. These traditional stoves mostly have low thermal efficiency, and as a result, more biomass fuels are burnt during smoking and cooking thus resulting in a high concentration of pollutants. High levels of smoke from cooking indoors with solid fuels commonly contain up to 1000 mg/m^3^ of particulate matter. In addition to particulate matter, nitrogen oxides, carbon monoxide, formaldehyde, and several toxic organic compounds such as polycyclic aromatic hydrocarbons are present in biomass smoke [10]. The concentrations of intake pollutants in solid biomass fuel users are usually higher as compared to the concentrations of intake pollutants among other forms of fuel users. This is largely due to the route of exposure to solid biomass fuel which is mostly through direct inhalation.

In rural areas of less developed countries, women often spend a lot of time cooking and therefore have a higher exposure duration to smoke emanating from solid fuel combustion [11]. The potential for exposure to children may further increase due to the common cultural practices in most rural areas where women usually carry infants and toddlers on their backs while cooking. Children with pneumonia who are exposed to biomass smoke suffer the most when it comes to household air pollution, which consequently leads to premature deaths in children. The highest estimated burden of pollution in adults is mostly those associated with cardiovascular diseases [12]. However, chronic obstructive pulmonary diseases and lung cancers could also be a major cause of disability and premature death in women who primarily engage in cooking activities in developing countries. In terms of emissions of particulate matter and other polluting gases, the Combustion of wood and other biomass is qualitatively similar to the burning of tobacco [12]. The process through which solid biomass fuel smoke adversely affects human health is thus likely to be similar to those involved in tobacco smoke. This could also mean that the pathways involved in solid fuel smoke-induced lung carcinogenesis are probably similar to that of tobacco smoke-induced lung cancers [13].

Indoor air pollution associated with the combustion of solid fuels is a major contributor to respiratory diseases. Indoor air pollution cause between 1.6 and 2 million deaths each year in developing countries [12]. Murray and Lopez [14] indicated that the burning of wood, dried animal compost and other biomass fuel such as twigs and shrubs is the chief cause of acute respiratory infections such as pneumonia which leads to the death of children under 5 years in most developing countries. Poverty-related risk factors, including water quality, hygiene and household air pollution from the use of solid fuels, account for a large proportion of under 5 years deaths [15]. Among children lower than 5 years of age, 3–5 million deaths have been attributed to acute respiratory infections yearly [16, 17].

Biomass fuel produces different chemical compounds of which most are easily inhaled due to their size. These chemicals emitted into the atmosphere through the incomplete burning of biomass fuel leads to the emission of air pollutants like carbon monoxide, nitrogen dioxide, chlorinated dioxins, arsenic, lead, fluorine and vanadium which are all noxious to human health [18]. Several studies have also shown that household burning of solid biomass fuels can be associated with the emission of many metals that exceed acceptable limits thereby posing threat to human health [19–21]. In as much as the amount and the kind of chemical substances present in the smoke of solid biomass fuel depend on the moisture content of the biomass fuel and the source of the biomass, the chemical composition of the smoke from these biomass fuels is affected by the status of the fire and thermal efficiency of the stoves [22]. These factors play an important role in determining the severity of pollution from biomass fuels. Trace metals present in wood smoke can induce oxidative stress through the formation of reactive oxygen species in the form of superoxide ions, hydrogen peroxide, and hydroxyl radicals which subsequently can lead to DNA damage [21, 23].

Exposure to indoor air pollution resulting from the incomplete burning of biomass fuels has been linked to many diseases [24]. Pneumonia which causes most deaths in young children, particularly those under 5 is associated with indoor air pollution resulting from the incomplete combustion of solid fuels [11, 25]. A health risk assessment conducted by De Oliveira et al. [26] for PM_2.5_ from the burning of solid fuel among children indicated that children were exposed to high levels of PM_2.5_, resulting in high toxicological risk. Balmes [12] and Embiale et al. [12, 22] also show in their study that solid biomass fuel users are exposed to high concentrations of PM_2.5_ and total organic volatile matter which can lead to early manifestation of several respirable diseases and pose a higher risk to those who are already associated with other respirable diseases. Other diseases that have been linked to smoke from biomass fuel include acute and chronic respiratory diseases such as asthma. However, few studies have been conducted on the effect of smoke generated in the fish smoking environment where mostly inappropriate stoves are used on human health.

In Abuesi, a major fishing community in the Western Region of Ghana, for instance, fish smoking is the commonest fish preservation method used and most women who are fish smokers use outdated smoking stoves with the use of biomass fuel such as wood as the main source of energy. These women mostly work carrying their young children at their backs and spend several hours smoking. Not enough studies have been conducted on the health hazards related to air pollution from the burning of biomass fuel for the smoking of fish with the use of outdated smoking stoves and the outcome of this study will help provide information on levels of harmful chemicals emitted into the environment, those most affected by these chemicals and the associated human health risk. Although emerging evidence, across the world, suggests that the use of unprocessed solid fuel for cooking is related to several blinding and painful eye conditions [27–29], there has been little research in developing countries such as Ghana to ascertain this link. Besides, there is a paucity of research on this environmental risk factor in the context of fish smoking. This study therefore, sought to examine the concentration levels of particulate matter (PM_2.5_) and volatile organic compounds (VOCs) from biomass combustion for fish smoking while monitoring key environmental conditions such as temperature and humidity. The study further sought to investigate the PM_2.5_ and VOC-related health symptoms experienced by the fish smokers in the study area.

## Materials and methods

### Research design

Concurrent mixed methods were used for this study. This study design employs multiple means of data collection such as surveys or interviews and data collection from field sampling at the same time. Field experimentation was employed by assessing the concentration levels of PM_2.5_ and VOCs from biomass combustion for the smoking of fish. Key environmental conditions such as temperature and humidity were also monitored. The Air Quality Meter (UNI-T, UT338C) was used for this purpose. A structured questionnaire was also used to gather information on the PM_2.5_ and VOC-related health symptoms experienced by the fish smokers.

### Study area

The study area is Abuesi; a local fishing community well noted for fish smoking. The natives especially the men are mostly fishermen and the women are noted for smoking fish as their major occupation. Abuesi is located in the Shama district in the Western Region of Ghana and it is 20 km from the Western Regional capital, Takoradi. The Town is about 5 km from Shama Junction and it is bordered on the east by Shama, on the north by Inchaban, the west by Injreisia and on the south by the Atlantic Ocean. Its coordinates are 4°58’60" N and 1°37’60" W [30].

#### Climate and topography

Abuesi lies within the tropical climate zone and experiences two rainy seasons. The major season is between March and July and the minor rainy season occurs between September and November. The highest rainfall is 170 mm, while the lowest is 100 mm. The average annual rainfall is 129.53 mm. The district has a relatively mild temperature ranging between 22 ºC and 28 ºC. In terms of humidity, precipitation occurs mainly from March to July when close to 70% of the rainfall takes place. The landscape of Abuesi is generally undulating and has faulty shelves and sandstones latent on a hard basement of granite, gneiss and schist [30].

### Fish smoking process

**Fig 1:**
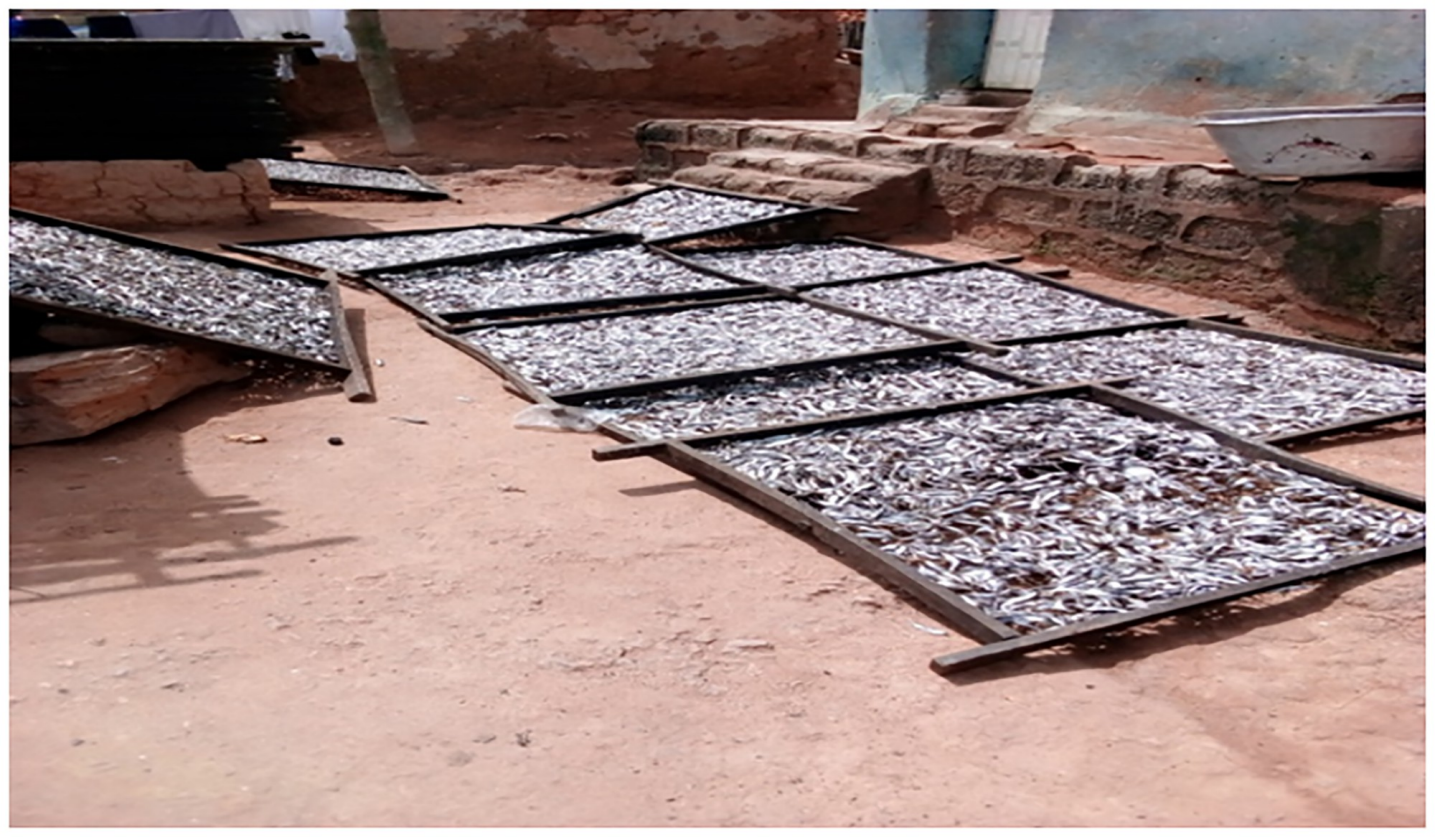
Drying of fish. Washed fish arranged on metal wiring nets with holes and dried under the sun for 20–30 min.

**Fig 2:**
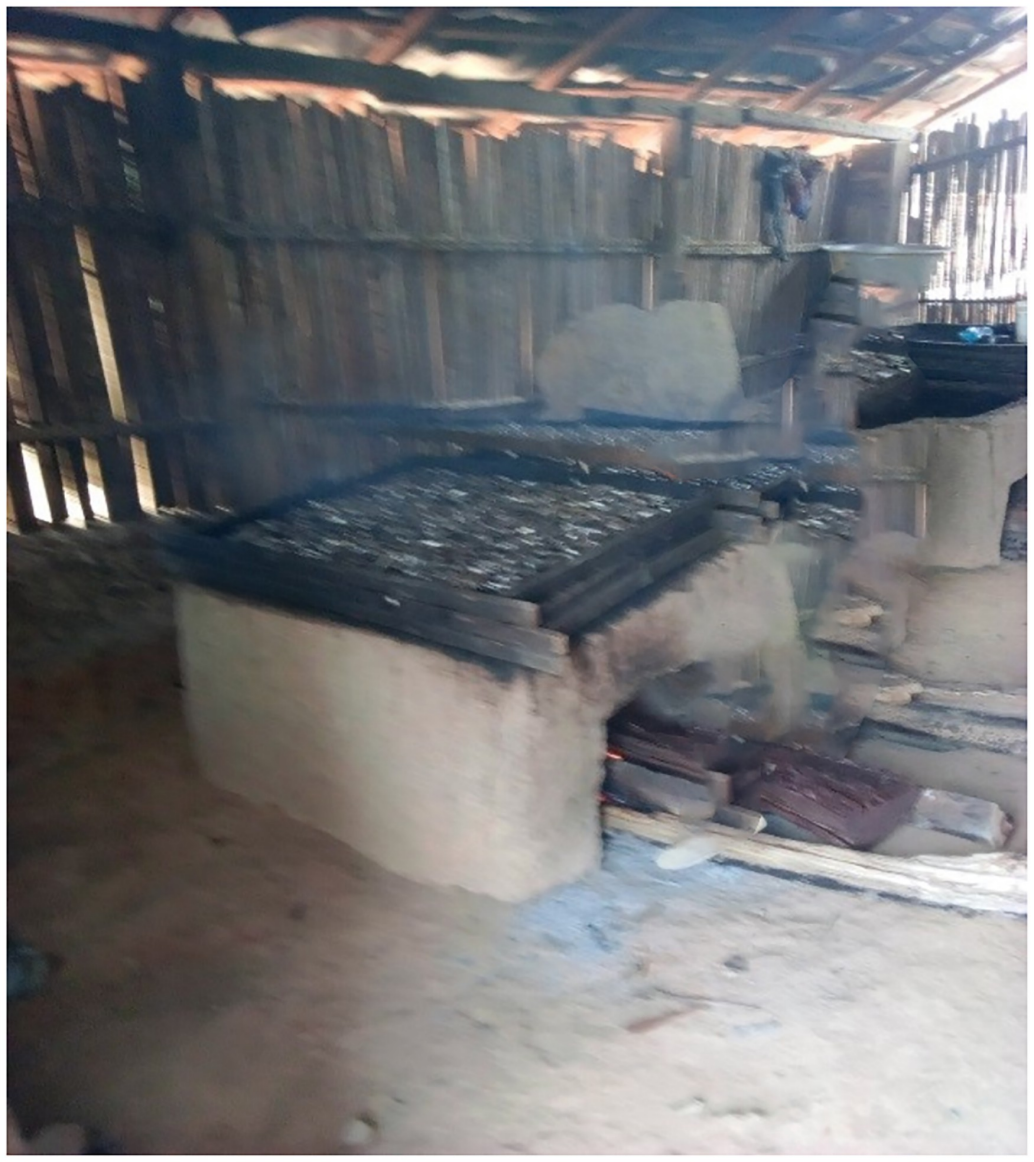
Smoking of fish. Dried fish smoked on mud ovens with fuel wood burnt inside the oven but under the metal net.

Fish are usually smoked in traditional smokehouses which are built normally with wood and/or bricks as seen in Figs 1 and 2. Smokehouses are usually located a few hours away from the community while others are located inside the community. Fish smokers mostly buy the fish at the bank of the sea or buy them from fish sellers who make them available to the fish smokers at their smokehouses. The fish are washed and arranged on metal wiring nets with holes to help get rid of water by drying it under the sun for 20–30 min. The fish are then smoked on mud ovens with fuel wood burnt inside the oven but under the net. Two main fishing seasons are observed in this region, that is, the lean season which starts in April and ends in the middle of July and the bumper season which also starts from August to October and ends in December.

### Data collection instruments and sampling procedures

#### PM_2.5_ and VOC monitoring.

**Fig 3:**
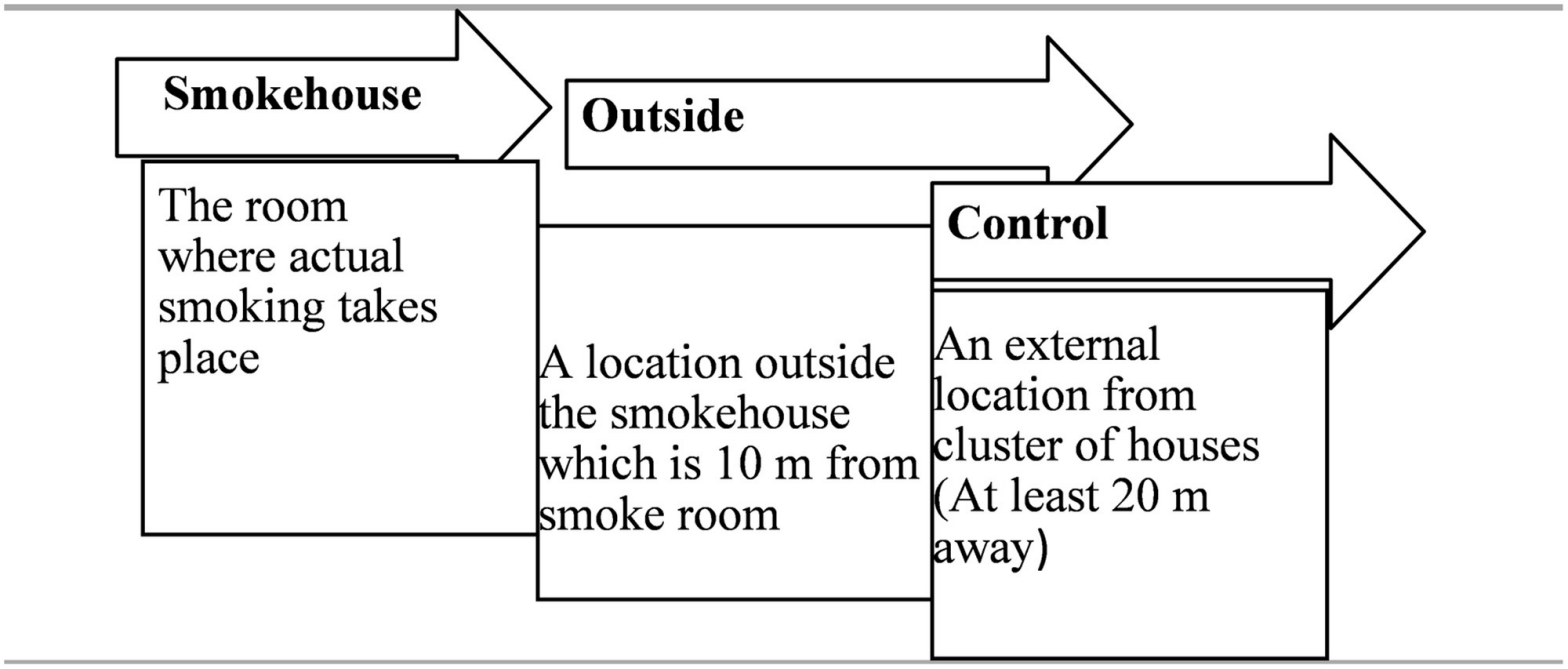
Sampling procedures for PM_2.5_ and VOC. A schematic diagram representing how the smokehouses, outside environments and control locations were selected and sampled for the study.

Data on PM_2.5_ and VOCs were collected using an Air Quality Meter (UNI-T, UT338C, China). Sixty (60) smokehouses were randomly selected and monitored for PM_2.5_ and VOC for 3 months depending on the day and time smoking was done by the selected participants. All enclosed smokehouses within the study area were given numbers from 1 to 243 to avoid double monitoring. A systematic sampling approach was then employed to choose 60 out of the total number. This was done by systematically counting and selecting every 4th smokehouse within the clustered areas where these smokehouses were located. Further, the names of the owners of these smokehouses were listed and follow-up monitoring was conducted. Monitoring readings were then taken from three locations; 1 = inside the smokehouse (indoor); 2 = outside the smokehouse (outdoor), which is 10 m from the smokehouse; and the control, which is 20 m away from the cluster of smokehouses (external environment). The 10 m interval was used to determine the differences in the distribution levels of PM_2.5_ and VOCs. A description of the study procedures is presented in Fig 3. The duration of the PM_2.5_ and VOCs monitoring covered three months. Readings were taken only on working days taking into account the time smoking was done. PM_2.5_ and VOCs monitoring were conducted at least 3 times a week for 4–5 h a day. Fish smoking normally starts after noon since the fish are mostly dried in a sieve under the sun before smoking.

#### Survey

A structured questionnaire was used to solicit for information to investigate the fish smoking-related health symptoms experienced by the fish smokers and also the relationship between their demographic factors and these health symptoms. Information on age, number of years spent smoking, type of biomass fuel used and education were all obtained with the aforementioned data collection tool. The target population included adult fish smokers who smoke fish in smokehouses and have been in the smoking business for a year or more. Participants were selected based on their availability to respond to questions as well as their willingness to participate in the study. With this approach, 434 fish smokers were selected at random and interviewed. This study was conducted from January 2017 to June 2017 among fish smokers who have been in the smoking business for a year or more and smoke fish in old smokehouses at Abuesi in the western region.

#### Reliability and validity

To ensure that the questionnaire used for the data collection is reliable, the questionnaire was pre-tested among 15 fish smokers. Identified errors such as difficulties in the interpretation of the questions were worked on by using simple and meaningful sentences. The exercise, therefore, allowed for the restructuring of the final research instrument before administration.

Air Quality Meter (UNI-T, UT338C) which is a hand-held digital device used for taking PM_2.5_ and VOC levels was calibrated by restarting using the user manual before taking it to the field to help avoid errors.

### Ethical consideration

Ethical approval was sought from the University of Cape Coast Ethical Review Board. Permission was also sought from the Chief of the community. Participants were not forced to participate in the study or financially induced to participate in this study and therefore could choose to either participate or not. They were informed that the information provided will contribute to the overall knowledge about biomass exposure and health problems among traditional fish smokers. Verbal consent was sought from the adult fish smokers while parental or guardian consent and assistance were sought for participants below 18 years. A record of the consent was kept as an audio/voice recording.

### Data processing and analysis

Data obtained from the field was analysed using the Statistical Package for Social Sciences (SPSS version 20) and Excel 10 (Microsoft). The software was mainly used to provide descriptive statistics of the various variables studied. The results were presented in tables and graphs. Parametric tests such as correlation and non-parametric tests such as Pearson’s chi-squared test and the Kruskal-Wallis test were used to find out the relationships between variables measured. The effect of socio-demographic variables (age, years of experience in smoking fish, education, etc.) on the fish mongers’ likelihood of reporting specific health outcomes (eye infections/irritation, coughing, etc.) was analysed using odds ratios (OR).

## Results

### Demographic characteristics of the study population

The characteristics of the study population are shown in Table 1. Most of the participants were above 44 years (38%), married (76%), and with no formal education (58%). The majority of these fish smokers have been in the smoking business for more than 9 years (75%), Christians (80%), and work for more than 3 h (94%) each smoking day. Most of the participants (70%) were aware of the health implications associated with exposure to fish smoking and have at least suffered health hazards like severe headaches, coughing and eye problems.

**Table 1:** Summary characteristics of respondents (N = 434).

Variables	Frequency	Percentage (%)
**Age**		
15–20 years	6	1
21–26 years	39	9
27–32 years	77	18
33–38 years	81	19
39–44 years	67	15
Above 44 years	163	38
**Marital Status**		
Single	39	9
Married	330	76
Divorced	44	10
Widow	21	5
**Highest Educational attainment**		
Non Formal Education	220	51
Primary Education	133	30
Junior High/ Middle School	56	13
Senior High/ Vocational Training School	20	5
Tertiary	5	1
**Number of Years in fish Smoking**		
1–4 years	50	12
5–8 years	58	13
9 years or above	326	75
**Religious Affiliation**		
Christian	347	80
Muslim	83	19
Traditional	4	1

### Levels of PM_2.5_, VOCs and key environmental factors associated with fish smoking

Table 2 presents the correlation coefficients of mean levels of PM_2.5_, VOCs, temperature and relative humidity levels. PM_2.5_ inside the smokehouse had a significant correlation (r = 0.461, p < 0.05) with VOCs inside the smokehouse. PM_2.5_ outside correlated strongly (r = 0.620, p<0.05) with VOCs outside and with temperature (r = -0.386, p < 0. 05) inside the smokehouse. Similarly, PM_2.5_ for the control location had a significant correlation with VOCs (r = 0.356, p< 0.05) outside and also with VOCs (r = 0.627, p < 0.05) from the control locations and lastly with the temperature outside (r = 0.373, p < 0.05). VOCs outside had a strong correlation with VOCs for the control location (r = -0.631, p < 0.05). Likewise, the temperature inside the smokehouse correlated with relative humidity inside the smokehouse (r = 0.380, p < 0.05). Finally, relative humidity inside correlated with relative humidity outside (r = 0.383, p < 0.05) while relative humidity outside correlated with relative humidity from the control locations strongly (r = 0.737, p< 0.05).

**Table 2:** Correlation matrix of environmental variables studied.

	**PM Inside**	**PM Outside**	**PM Control**	**VOCs Inside**	**VOCs Outside**	**VOCs Control**	**Temp. Inside**	**Temp. Outside**	**Temp. Control**	**Rel. Hum Inside**	**Rel. Hum Outside**	**Rel. Hum Control**
**PM Inside**	**1**											
**PM Outside**	**-0.024**	**1**										
**PM Control**	**-0.038**	.334**	**1**									
**VOC Inside**	.461**	**-0.005**	**0.132**	**1**								
**VOC Outside**	**0.029**	.620**	.356**	**0.247**	**1**							
**VOC Control**	**-0.063**	.417**	.627**	**0.209**	.631**	**1**						
**Temperature Inside**	**0.057**	-.386**	-.260*	**-0.057**	-.316*	-.316*	**1**					
**Temperature Outside**	**0.051**	-.304*	-.373**	**-0.14**	**-0.248**	**-0.238**	.862**	**1**				
**Temp. Control**	**-0.137**	**-0.191**	**-0.12**	**-0.16**	**-0.037**	**-0.068**	.324*	.297*	**1**			
**Rel. Hum Inside**	**0.189**	**-0.138**	**-0.121**	**-0.085**	**-0.241**	**-0.139**	.380**	**0.249**	.295*	**1**		
**Rel. Hum Outside**	**0.135**	**-0.15**	**0.078**	**-0.034**	**-0.123**	**0.038**	**0.254**	**0.163**	**0.024**	.383**	**1**	
**Rel. Hum Control**	**0.077**	**-0.231**	**0.048**	**0.009**	**-0.098**	**-0.075**	**0.157**	**0.07**	**-0.016**	.264*	.737**	**1**

Regression analysis was run to assess the influence of key environmental factors studied (temperature and relative humidity) on key air pollutants (PM_2.5_ and VOCs) over the study period. There was a positive relationship between PM_2.5_ inside and relative humidity inside (R^2^ = 0.0305, p<0.05). This meant that a unit change in relative humidity could cause a corresponding change of about 3% in PM_2.5_ inside the smoke room. There was however no relationship between PM_2.5_ inside and temperature inside. A significant negative relationship was found between VOCs inside and relative humidity inside (R^2^ = 0.0073, p< 0.05). The regression coefficient indicated that a change in relative humidity inside is likely to cause a corresponding decrease in VOCs. Further, a negative relationship between the temperature inside and VOCs inside (R = 0.0032, p<0.05) was found. The coefficient indicates that a unit increase in temperature will cause a corresponding decrease in VOCs inside.

Table 3 presents the mean levels of the variables studied in all locations. The mean levels of PM_2.5_ inside the smokehouse were 321.58 μg/m^3^ with a minimum and maximum value of 164 μg/m^3^ and 493 μg/m^3^ respectively at a standard deviation of 90.53. The range of PM_2.5_ outside recorded was 155 μg/m^3^ and 255 μg/m^3^ with a mean value of 178.60 μg/m^3^. Whereas the PM_2.5_ recorded at the control point also ranged from 42 μg/m^3^ to 266 μg/m^3^ with a mean of 175.80 μg/m^3^. Comparing the mean values of PM_2.5_ in the various locations revealed that, PM_2.5_ found indoors and outdoors were significantly different (t = 11.9, p < 0.05). Also, PM_2.5_ indoor and control were significantly different (t = 12.0, p < 0.05). However, the concentrations of PM_2.5_ found in the outdoor and control locations were not significantly different (t = 0.69, p > 0.05). The outcomes of the results using the t-test imply that the mean concentration of PM_2.5_ recorded inside the smokehouse and outside locations were significantly different indicating a possible differential impact.

**Table 3:** Descriptive statistics of variables.

Variables	Minimum	Maximum	Mean	Std. Deviation
PM_2.5_ Inside	164	493	321.58	90.53
PM_2.5_ Outside	155	255	178.6	19.84
PM_2.5_ Control	42	266	175.82	23.74
VOC Inside	3.9	9.9	8.56	1.64
VOC Outside	1.9	9.9	5.25	1.70
VOC Control	1.6	9.9	4.38	1.65
Temperature Inside	30	36	31.85	1.44
Temperature Outside	30	35	31.78	1.33
Temperature Control	5	38	31.47	3.86
Relative Humidity Inside	0	99	63.8	41.09
Relative Humidity Outside	0	99	80.97	25.53
Relative Humidity Control	1	99	83.88	21.09

The mean levels of VOCs found inside the smokehouse were 8.56 mg/m^3^ with minimum and maximum values ranging from 3.90 mg/m^3^ to 9.90 mg/m^3^. The mean VOC levels recorded outside were found to be lower than VOCs inside (5.25 mg/m^3^) with minimum and maximum values recorded at 1.90 mg/m^3^ and 9.90 mg/m^3^ whilst VOC control levels ranged from 1.60 mg/m^3^ and 9.90 mg/m^3^ with 4.38 mg/m^3^ as the mean. Similar to PM_2.5_, the findings of this study revealed a significant difference in the levels of VOCs between the indoor and outdoor locations (t = 10.8, p < 0.05), indoor and control (t = 13.9, p < 0.05) as well as VOCs of outdoor and control (t = 2.83, p < 0.05), respectively.

Temperature gradients also fluctuated over the 3-month study period. The mean temperature levels recorded inside were 31.85 °C with minimum and maximum values ranging from 30 °C and 36 °C. Mean temperature levels outside the smokehouse rose relatively to 31.78 °C with mean readings recorded between 30 °C to 35 °C. In the control regions, the mean temperature levels recorded were 31.98 °C and with a range from 5 °C to 38 °C as the mean. Relative humidity values were also found to fluctuate from locations where readings were taken over time. For instance, the mean relative humidity level recorded inside was 63.80% with minimum and maximum values ranging from 0% and 99%. The relative humidity levels recorded outside the smokehouse also ranged from 0% to 99% with a mean of 80.97% while the control levels ranged from 1% to 99% with 83.88% as the mean, respectively.

#### Temporal distribution of PM_2.5_ and VOCs in indoor and outdoor environments.

Kruskal-Wallis test was used to test for the significance of the differences in the temporal variation in the air pollutants and environmental conditions inside and outside the smokehouse. It was observed that the relative humidity measured inside the 60 selected smokehouses was the same across the 3 months of monitoring, meaning there was no significant difference or variations in relative humidity measured inside the 60 selected smokehouses. Relative humidity outside showed significant differences indicating that there are variations in the distribution of relative humidity outside across the three months. Also, the relative humidity control showed significant differences indicating that there are differences in the distribution of relative humidity across the three months for the control locations. Differences in temperature inside the smokehouse, outside the smokehouse and temperature control were all significant indicating that there are differences in the distribution of temperature across the three months.

The differences in the PM_2.5_ levels recorded outside the smokehouse and PM_2.5_ levels recorded at the control points in all the sixty locations were significant, indicating that there are differences or variations in the distribution of PM_2.5_ inside and outside the smokehouse across the three months. PM_2.5_ inside the smokehouse and PM_2.5_ outside the smokehouse however did not show a significant difference. VOC levels recorded inside the smokehouse did not show significant differences in their distribution across the 3 months. However, VOCs outside and VOC control showed significant differences indicating that there are significant differences in the distribution of VOCs across the study months.

### Self-reported health symptoms associated with fish smoking

Pearson’s chi-square test of independence revealed that there was no statistically significant difference between fish smoking, educational attainment (χ2 (4) = 1.08, P = 0.898), the number of years of experience in fish smoking (χ2 (2) = 2.84, P = 0.242), religion (χ2 (1) = 0.59, P = 0.444) and marital status (χ2 (3) = 5.42, P = 0.144). A statistically significant difference was found between fish smoking and the age of fish smokers (χ2 (5) = 11.51, P < 0.05). Table 4 depicts the self-reported health hazards identified during the survey. Three main health symptoms namely eye problems, cough and headaches were the most reported fish smoking-related health cases.

**Table 4:** Identified fish smoking-related health symptoms.

Variable	Eye problem (%)				Cough (%)				Headache (%)			
	No	Once	Twice	Several Times	No	Once	Twice	Several Times	No	Once	Twice	Several Times
**Age (Years)**	**P = 0.161**				**p = 0.154**				**P = 0.115**			
15–20	0	25	25	50	67	17	16	0	17	0	16	67
21–26	42	25	8	25	36	13	5	46	67	0	17	16
27–32	29	13	29	29	45	13	3	39	67	8	13	12
33–38	29	28	5	38	47	14	0	39	63	13	4	20
39–44	13	34	0	53	46	12	3	39	47	0	13	40
>44	24	11	14	51	36	9	2	53	36	7	8	49
**Educational Level**	**P = 0.731**				**P = 0.106**				**P = 0.205**			
No formal education	24	19	14	43	42	10	2	46	51	12	3	34
Primary	25	17	8	50	38	10	4	48	56	3	18	23
Junior High/Middle school	28	28	22	22	37	11	4	48	42	0	21	37
Senior High/Vocational	40	20	0	40	32	25	0	43	60	0	0	40
Tertiary	100	0	0	0	100	0	0	0	0	0	0	100
**Years spent smoking fish**	P<0.043*				**P = 0.162**				**P = 0.452**			
1–4 years	38	19	12	31	52	12	4	32	50	14	11	25
5–8 years	45	23	5	27	52	12	3	33	50	9	14	27
9 years or more	15	19	16	50	39	11	2	48	51	3	9	37

**Fig 4:**
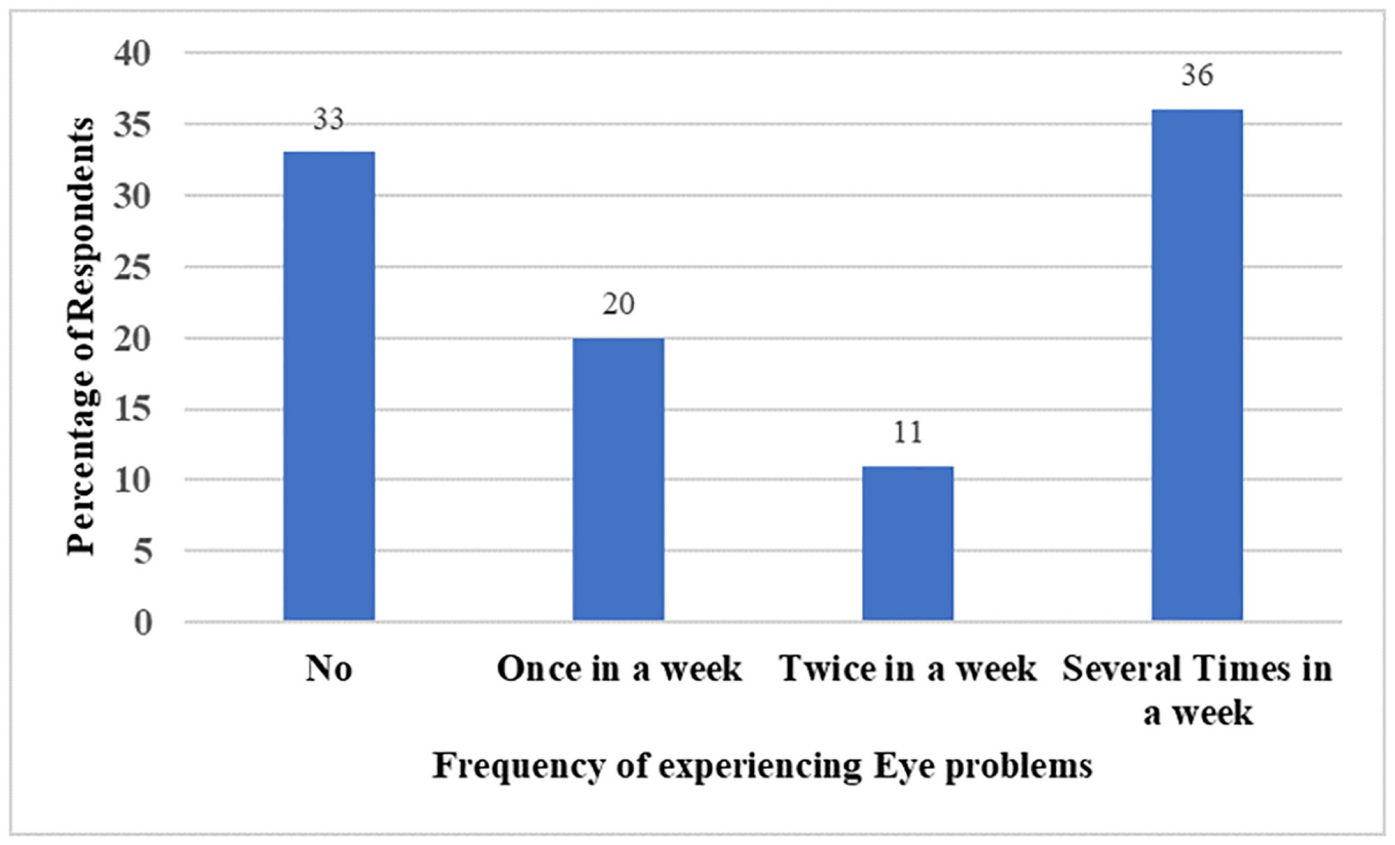
Association between fish smoking and eye irritation or infection.

From Fig 4, 33% of the fish smokers reported that they did not experience eye irritation or infection while the remaining 67% reported experiencing eye irritation once, twice, and several times a week. The result also showed that there was a statistically significant association between fish smoking and eye irritation (χ2 (3) = 44.75, P < 0.05). The Cramer’s V of 0.61 indicated that the association between them was strong.

**Fig 5:**
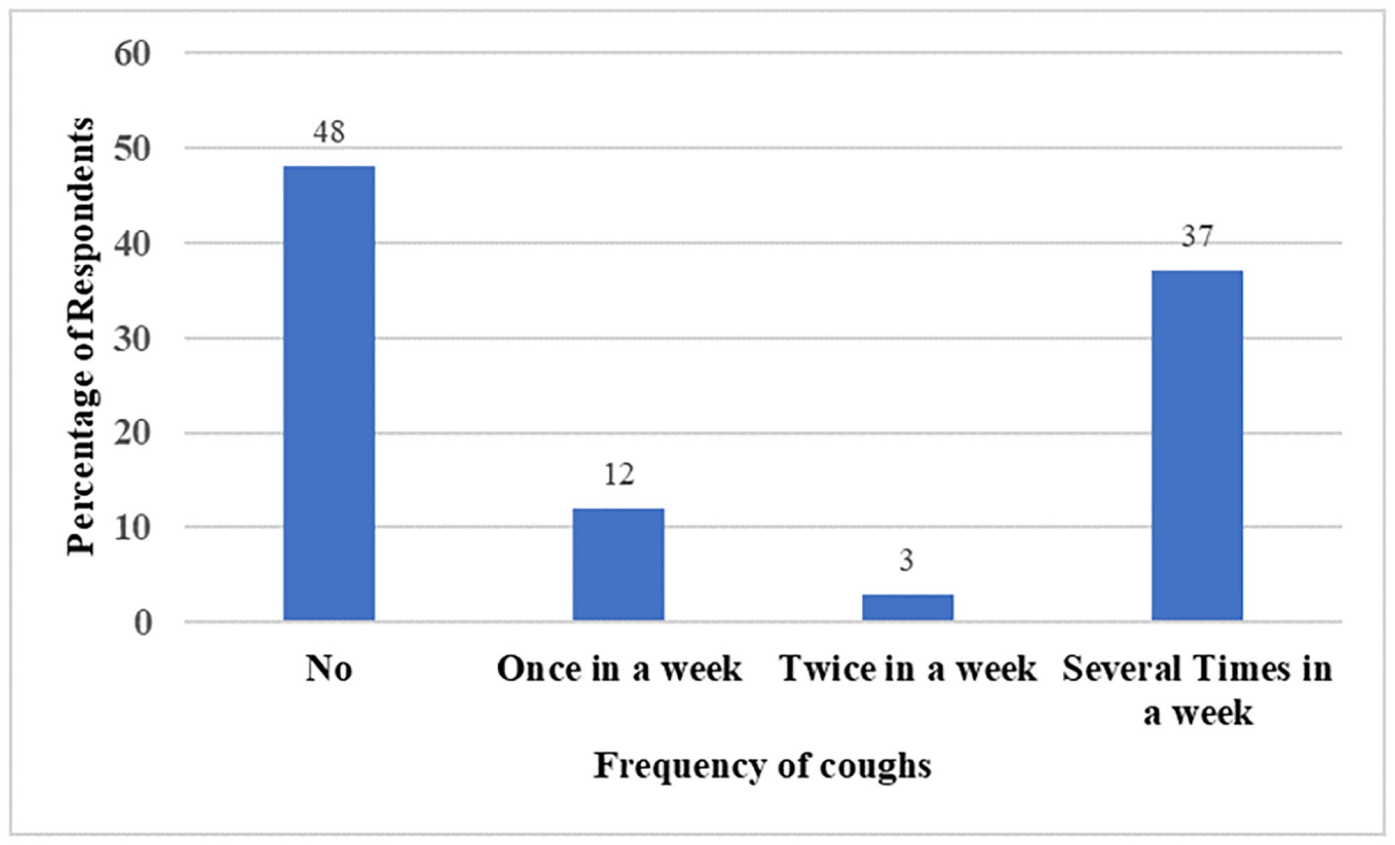
Association between fish smoking and cough.

From Fig 5, 48% of the fish smokers reported that they did not experience a cough while a greater percentage of them reported experiencing a cough once, twice, and several times a week. The result also showed that there was a statistically significant association between fish smoking and cough (χ2 (3) = 8.71, P< 0.05). The Cramer’s V of 0.26 indicated that the association between them was strong.

**Fig 6:**
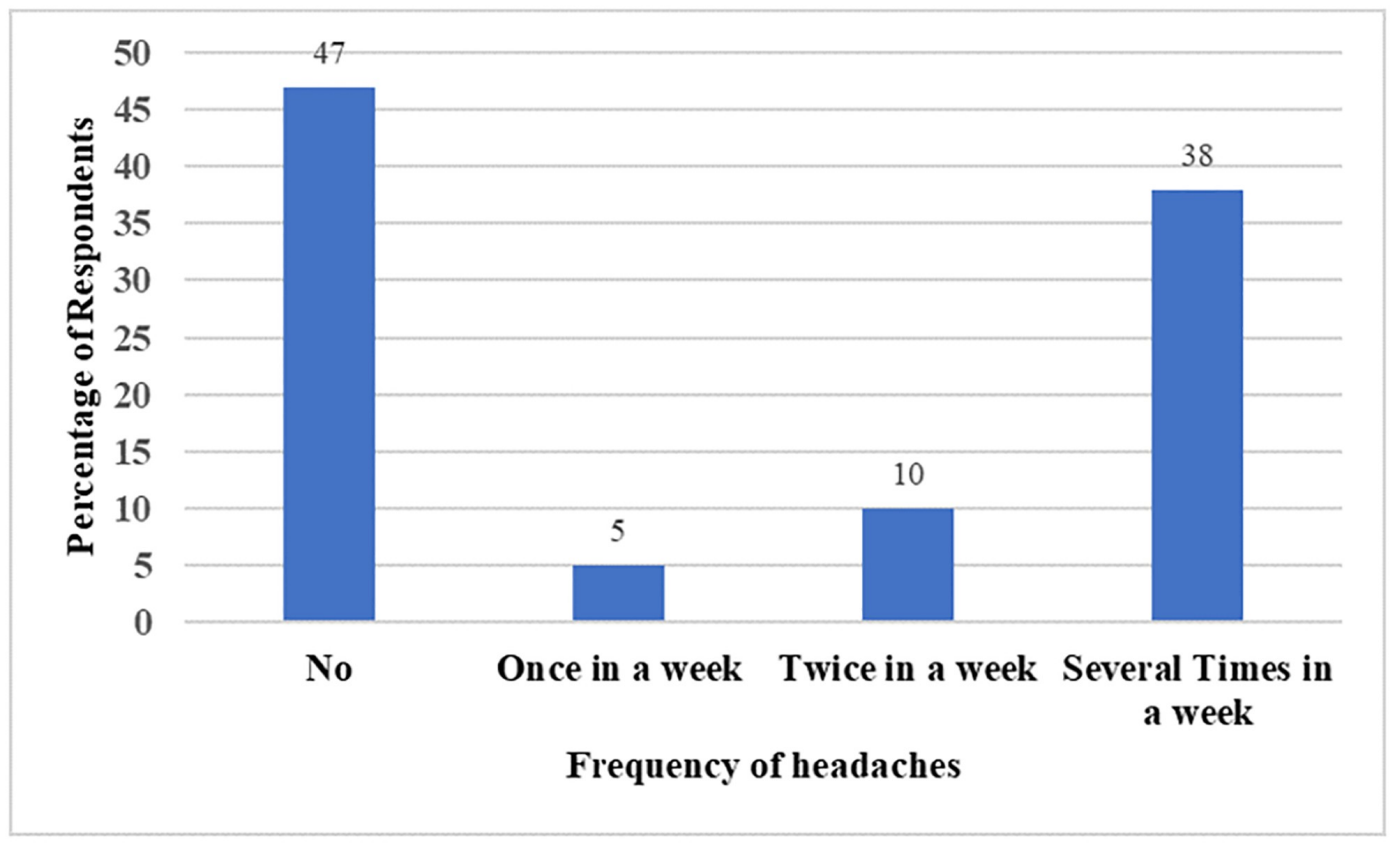
Association between fish smoking and headache.

From Fig 6, 47% of the fish smokers reported they experienced no headaches while the remaining 53% reported experiencing headaches once, twice and several times a week. There was a statistically significant association between fish smoking and headache (χ2 (3) = 9.42, P< 0.05). The Cramér’s V of 0.28 indicates that the association between them is moderately strong.

**Fig 7:**
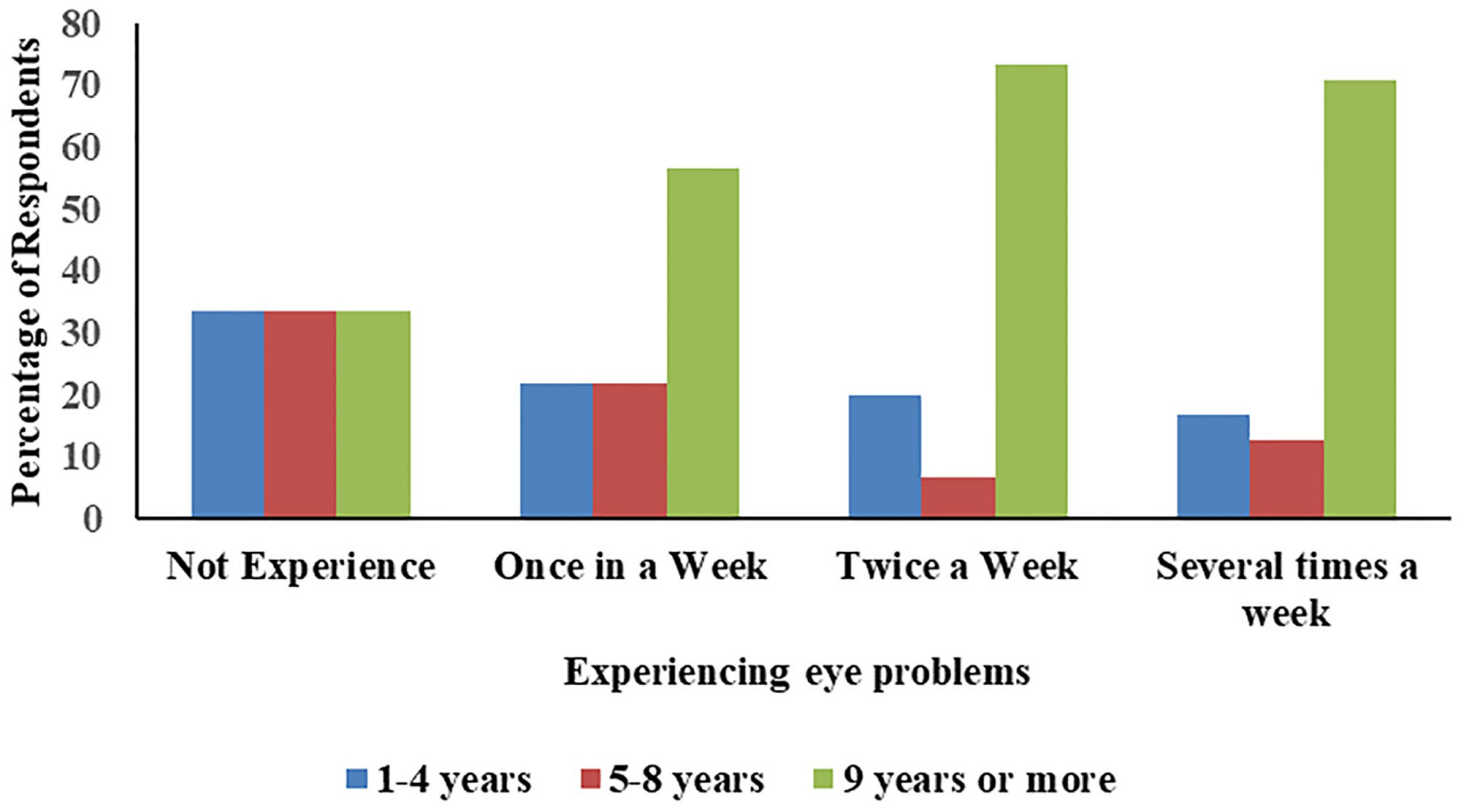
Frequency of eye problems and the number of years spent smoking.

The findings further revealed that the age of the respondents and their educational levels had no significant effect on all three reported cases. However, it was observed that the prevalence of eye-related problems was influenced by the number of years the women had been in the fish smoking business. Fig 7 shows the association between the frequency of reported eye problems and the number of years spent smoking fish. Fish smokers who had worked for more than 9 years reported most eye infections or irritations. Seventy-one percent of them reported experiencing eye problems several times a week, 73% reported that they experienced eye infections twice a week and lastly 55% reported experiencing eye infections or problems once a week. The Chi-square test indicated that there is a statistically significant association between eye infection or irritation and the number of years spent smoking fish (χ2 (6) = 13.02, P <0.05). The Cramér’s V of 0.24 indicates that the association between them is moderately strong.

As shown in Table 5, a logistic regression analysis was further run to find the likelihood of reporting eye problems among the fish smokers based on the number of years spent smoking fish. Using 1–4 years as a reference, it was found that fish smokers who have engaged in fish smoking for 9 years or more were 2.7 times more likely to complain of experiencing eye problems as compared to those who have been smoking for lesser years.

**Table 5:** Relationship between exposure to smoke and eye problems.

Frequency of experience of eye disease	N	OR	Conf. Interval
<4 years	50		
5–8 years	58	0.721	0.231 2.246
9 years or more	326	2.723*	1.138 6.513

## Discussion

Particulate matter is a complex mixture of particles of several sizes and chemical compositions. Depending on the aerodynamic diameter of the particles, the term PM_10_ is used to indicate particles with a diameter of up to 10 μm and PM_2.5_ for particles with a diameter of up to 2.5 μm. Within the PM_2.5_ category, there is a distinction between fine (≤ 2.5 μm) and ultra-fine (<0.1 μm) particles. The largest particles are the coarse fractions that are mechanically produced by the break-up of larger solid particles. These particles include windblown dust from agricultural processes, soil, unpaved roads, or mining operations. Traffic produces road dust suspended from the road surface. Near the coast, evaporation of sea spray can produce large particles as well. The brakes of vehicles produce particles in somewhat smaller sizes. Fine fractions are largely formed from gases. The smallest particles, less than 0.1μm, are formed by condensation of substances or combustion. Hence, the size and composition of particles in the ambient air vary.

This study measured and compared the PM_2.5_ inside the smokehouses, outside the smokehouses and in an area used as the control point. As seen in this study, the highest mean values were found inside the smokehouses followed by outdoor locations and control locations respectively. All the PM_2.5_ values obtained were above both the WHO (25 μm) and Ghana Environmental Protection Agency (35 μm) threshold for indoor air quality. The PM_2.5_ recorded in all locations was about 3–6 times higher than the permissible levels which shows the extent to which fish smokers have been exposed to these harmful emissions since they spend more than 4 h a day inside their smoke rooms. These results were consistent with that of Kim et al. [31] who studied the transported vs. local contributions from secondary and biomass-burning sources to PM_2.5_. Further analysis also suggested significant differences in the concentrations of PM_2.5_ inside and outside the smokehouses, inside the smokehouses and control locations but no significant differences were found in the measured concentrations of PM_2.5_ in the outdoor and control locations. This trend also suggests that the presence of PM_2.5_ were distributed evenly in the environment of the smoking house. This study also found that there is a statistically significant difference in the PM_2.5_ levels in indoor and outdoor environments. This observation is also in line with the work of Ouyang [32]. The similarities in PM_2.5_ between indoor environments and outdoor locations might be caused by environmental conditions such as wind directions, time and topography. Based on this result, it is believed that women inside the smoking house are likely to experience the negative outcomes of exposure to high concentrations of PM_2.5_.

Particles in the atmosphere have received much interest in the last decade because increasing epidemiological and experimental evidence reflects a negative impact on human health and the environment [33, 34]. To investigate the health effects of PM, it is significant to take particle size, chemical composition and concentrations into account, since these metrics reflect the biological mechanism through which particle pollution is assumed to cause health effects [35]. PM arrives in several places in our airways and lungs through inhalation. Particles up to PM_10_ are called inhalable particles, ranging from particles with aerodynamic diameters below 0.1μm to 10μm [36]. Epidemiological studies reflect that particles smaller than 10 μm, can penetrate the airways by inhalation and penetrate deeper into our respiratory system than larger particles causing severe health hazards and even premature mortality [37]. Short-term effects of people exposed to PM_2.5_ may include inflammatory reactions in the lung, respiratory symptoms, adverse effects on the cardiovascular system and increases in medication use, hospital admissions and premature mortality [38]. Long-term exposure to PM_2.5_ is also coherent with severe (chronic) health effects such as increases in lower respiratory symptoms and chronic obstructive pulmonary diseases, reduction in lung function in children and adults, and premature mortality. The reduction in life expectancy is mainly due to cardiopulmonary (affecting both the heart and the lungs) mortality and probably to lung cancer [39]. The exact long-term effects of exposure to PM are however still ambiguous, as a result of conflicting results in long-term exposure studies [40].

Apart from the fish smokers themselves, young children of fish smokers are also vulnerable to smoke-related diseases. Children, infants, and fetuses differ strongly from adults in toxico-kinetic processes, e.g., lower body weight, the higher relative weight of the liver, the higher ratio between the body surface and body weight, smaller lung calibre, higher particle deposition in the respiratory tract, immature lungs, etc. Moreover, the lungs are completely formed at birth and remain immature until about 6 years [41]. According to Heinrich et al. [42], children are likely to experience health complications because the lungs are not fully developed, and toxins and other substances can pass more easily through the epithelial layer. Immature lung tissue makes children more vulnerable to (fine) PM.

Higher levels of VOCs were also recorded in most of the smokehouses. The mean VOC concentrations also reduced from inside the smokehouse to the immediate environment (outdoor) and the control locations. This study also found that there were statistically significant differences in the VOC levels in indoor and outdoor environments. This study demonstrated that fish smokers inside the smokehouse were exposed to higher VOC levels than people who had no direct contact with VOCs, similar observation was also reported by [43] where significant differences in VOCs levels were observed when assessing indoor and outdoor pollution levels. The concentrations of common VOCs in a given indoor environment are strongly related to the existence of emission sources and the efficiency of ventilation [44]. In this study, the emission source was mainly the combustion of fuel wood which produce relatively high concentrations of VOCs. In some cases, indoor VOC levels were extremely high, especially inside the smokehouse where smoking took place owing to low air exchange rates (AER) and poor ventilation [45]. Occupational exposure to VOCs is known to be very detrimental to human health. VOCs are ubiquitous in indoor environments. As reported by Huang et al. [45], inhalation of VOCs can cause irritation, difficulty in breathing, nausea, and damage to the central nervous system as well as other organs. These symptoms however may depend on the number of years victims have been exposed to fire or smoke and duration, age and experience gained in the fish smoking activity. This study found that there was an association between the number of years spent smoking fish and eye infections just as reported by [46].

The concentrations of the pollutants considered in this study could also be influenced by very key environmental factors such as temperature and humidity. Temperatures recorded throughout the study period ranged from 30 °C to 36 °C. The temperature values recorded were above recommended standards which are similar to the temperature levels recorded by [47]. The temperature values recorded were considered high, especially inside the smokehouse where the women spend about 90% of their time. On the other hand, humidity values fluctuated from sampling locations; inside, outside, and the control location respectively. Exposure to high temperatures can cause severe damage to the health of fish smokers. Indoor temperature and humidity are intertwined. Humidity levels are likely to drop in places or areas where temperatures are high. This scenario can explain the low humidity levels inside the smokehouses. The Canadian Standards Association recommends maintaining even office temperatures below approximately 26°C under conditions of typical relative humidity (CAN/CSA Z412-00). This suggests that the temperatures recorded between 30 °C and 36 °C can be considered unhealthy for fish smokers. Thermal comfort is important for health and well-being. An environment that is too warm can result in people feeling tired and uncomfortable which may impact their well-being and quality of life. However, a limitation of these standards is that thermal comfort is subjective and influenced by several factors, including clothing, activity level, metabolic rate, age and pre-existing medical conditions [48]. Also, thermal comfort temperatures may not reflect the temperatures at which people experience health impacts from heat. From a health perspective, it is important that any maximum indoor temperature standard considers health-based evidence rather than comfort thresholds alone. Reports by the World Health Organization (WHO) provide guidance on thermal comfort and suggest that there is a minimal health risk when indoor temperatures are between 18°C and 24°C.

This temperature range is based on research examining the health impacts of temperature for healthy individuals under conditions of appropriate clothing, humidity and air movement (i.e., not under high winds). The temperature range identified by the WHO has been acknowledged as the range within which health is optimally protected. The health impacts of extreme heat include heat stress, heat stroke, morbidity and mortality. People who experience high temperatures for prolonged periods, or are sensitive to heat, are the most vulnerable. This suggests that female fish smokers are also vulnerable to these health problems. There is still little evidence that specifically examines how indoor temperatures influence the health risk from the heat. Indoor and outdoor heat are related, although there are some differences [48]. Indoor temperature is modified by other factors related to building design and construction (e.g., quality of insulation), location of apartment unit (level of building, orientation), room type, and occupant behaviour (e.g., activity levels, density of occupants). Yet unlike outdoor temperature, indoor thermal conditions are not routinely monitored which makes studying the association between indoor temperature and health challenges important. However, a limited number of studies have focused specifically on the effects of high indoor temperatures, and demonstrate increased health impacts. For example, research from New York City, US found a greater proportion of emergency service distress calls for cases of respiratory illness in people in buildings with indoor temperatures above a 26°C threshold than in buildings with temperatures lower than 26°C [49].

A total of 434 female fish smokers were also drawn to participate in the study. The majority of the women were middle-aged women who were above 40 years old and had lived and depended on this economic activity for their livelihoods for a longer period. In Ghana, women are dominant in traditional fish processing and trade whether in large or small-scale operations [50]. This position is long-standing as indicated by their traditional roles, which, interestingly, include roles in coastal areas and inland markets (underlining the historical importance of the fish trade in Ghana) [51]. At landing centres, the sub-chief whose role dates to the early 20th century sets or influences the prices at which fish is sold from the boats and although these women traders may advance fishing trip costs to boat operators, which in turn will give them access to that boat’s catch, the price-setting mechanism means that they will not buy at the preferential prices that so often result from trader-credit relationships in fisheries [50]. There is some indication of the erosion of these systems (e.g., in situations where prices are negotiated on an individual basis), particularly in places with improved landing facilities notably where landing fees are payable—or where there are particularly successful and powerful fish mongers. In the Western Region of Ghana, smoking is the most common form of fish processing [51]. The women slowly smoke the fish on stacked racks, with relatively efficient use of fuel wood, producing a relatively evenly- smoked product [52]. The level of literacy among fish smokers is usually low. This study revealed that about 72% of the respondents had only completed primary school. This result confirms the report by the Ghana Statistical Service that formal education among most coastal dwellers of Ghana is low.

Firewood was the main source of energy used by fish smokers. The biofuels and smoking practices used were not different from those used by fish smokers in other coastal areas in the country [53]. Fish smoking is done at the artisanal level by women in coastal towns and villages and areas along rivers and the shores of Lakes. The reasons for smoking fish are varied but, as far as Ghana is concerned, the process has proved relevant to prolonging shelf life; enhancing taste and increasing utilization in food preparation; reducing waste at times of bumper catches; storing for the lean season, and thus increasing protein availability to people throughout the year. Therefore, depending on the purpose, fish smokers choose a type of biofuel that would be perfect for their work. The findings from this study are not different from those conducted in most developing countries across the globe. For instance, Stoner et al. [54] reported that close to 53% of the world’s population (around 3 billion people) use biomass fuels as their primary source of domestic energy for cooking, home heating, and light; ranging from nearly 9% in developed countries to more than 80% in China, India, and Sub-Saharan Africa. In the rural areas of Latin America, approximately 30 to 75% of households use biomass fuels for cooking. Wood is the biomass fuel most frequently used both as unprocessed wood and as charcoal, the latter having a far lower impact on indoor air pollution. In some regions, especially in sub-Saharan Africa, approximately 20% of the wood energy harvested is processed into charcoal and could reach 50% in some countries. The use of animal dung, crop residues, corncobs, and grass increases when the wood is scarce or the forests are situated far away from the community. The current socioeconomic situation in many developing countries suggests that the use of biomass fuels will continue in the coming decades. The total annual average of wood products used for fuel in developing countries increased by approximately 16.5% over the past years [55]. However, a significant number of these wood smoke constituents are known to be toxic or irritants for the respiratory system, including PM, carbon monoxide (CO), nitrogen and sulphur oxides (NO_2_, SO_2_), aldehydes (e.g., formaldehyde), polycyclic aromatic hydrocarbons (e.g., benzo- pyrene), VOCs, chlorinated dioxins, and free radicals. Many substances can act as primary pollutants, irritants, and co-carcinogenic compounds [56].

Health-related cases associated with the combustion of biofuels reported by fish smokers include eye irritation or infections, headaches and cough. Most of the reported health cases resulted from persistent exposure to smoke from firewood used by fish smokers. This observation confirms the self-reported symptoms associated with biomass fuel use reported by Walker et al. [57]. This study, however, found that the prevalence of health symptoms was influenced by factors such as age and the number of years of experience in fish smoking. With regards to the link between the number of years spent smoking fish and the frequency of self-reported eye problems, it was found that prolonged exposure to biofuels for smoking fish is likely to cause an eye infection. Eye irritation is mostly incurred in the form of tearing whiles smoking [58]. There was a massive exposure response between the number of years of smoking fish and chronic cough, eye problems, and other symptoms. A similar observation was made by [59]. This study showed that several factors in synergy increase health risks from indoor air pollution resulting from extensive use of biomass fuel for fish smoking. Consequently, the likelihood of fish smokers experiencing health symptoms in their lifetime is high [60].

## Conclusion

This study sought to assess the levels of particulate matter (PM_2.5_) and volatile organic compounds (VOCs) emanating from the burning of biomass fuel from the smoking of fish. It also sought to determine the proportion of fish smokers reporting health symptoms associated with exposure to these pollutants. PM_2.5_ monitored in all the locations were above the WHO (25 μm) and Ghana Environmental Protection Agency (35 μm) standards for indoor air quality especially those recorded in the smoking rooms. This indicates that fish smokers are exposed to high levels of PM which increases their risk of health outcomes as well as the young children who are mostly carried at the back of their mothers during smoking. The same could be said about those who are not directly involved in the smoking activity; they are exposed to the smoke due to the movement of the smoke emitted but the impact is not severe as compared to those who work inside the enclosed smokehouses with no appropriate ventilation and stay in the smokehouse for more than 4 h a day. The study also demonstrated that fish smokers inside the smokehouses or smoking rooms were exposed to higher VOC levels which are detrimental to their health since they spent more hours in the smoking rooms. Most fish smokers reported coughs, headaches and eye irritation or infections. It was also found that the number of years spent smoking fish is closely associated with the frequency of reported eye cases. Fish smokers who had been in the smoking business for more than 9 years reported the most eye problems. The Ministry of Fisheries and Aquaculture Development should conduct routine educational seminars and programs to help inform fish smokers about the human health risk associated with a high risk of exposure to smoke. Fish smokers should be educated on the need for fish smoking to be done in appropriate ventilated smokehouses. There is a need for further research to be conducted on PM_2.5_ and VOCs and their related human health exposure (non-respiratory symptoms) concerning fish smoking.

## Supporting information

S1 File (XLSX)
